# Preliminary studies on the molecular mechanism of intramuscular fat deposition in the longest dorsal muscle of sheep

**DOI:** 10.1186/s12864-024-10486-w

**Published:** 2024-06-12

**Authors:** Xuwen Shao, Xintan Lu, Xinming Sun, Huaizhi Jiang, Yang Chen

**Affiliations:** 1 College of Animal Science and Technology, Jilin Agricultural University, Changchun, 130118 China; 2 Key Laboratory of Livestock and Poultry Resources (Sheep & Goat) Evaluation and Utilization, Ministry of Agriculture and Rural Affairs, Changchun, China

**Keywords:** Small-tailed frigid sheep, Longest dorsal muscle, Intramuscular fat, Growth and development, Transcriptomics

## Abstract

**Background:**

Intramuscular fat content is an important index reflecting the quality of mutton, which directly affects the flavor and tenderness of mutton. Livestock and poultry intramuscular fat content is influenced by genetics, nutritional level, and environmental factors. Key regulatory factors play a crucial role in intramuscular fat deposition. However, there is a limited amount of research on the identification and function of key genes involved in intramuscular fat content deposition specifically in sheep.

**Results:**

Histological differences in the longest dorsal muscle of the small-tailed frigid sheep increased in diameter and decreased in several muscle fibers with increasing monthly age; The intramuscular fat content of the longest dorsal muscle of the small-tailed cold sheep varied with age, with a minimum of 1 month of age, a maximum of 6 months of age, and a minimum of 12 months of age. Transcriptomic sequencing and bioinformatics analysis revealed a large number of differential genes in the longest dorsal muscles of little-tailed billy goats of different months of age, which were enriched in multiple GO entries and KEGG pathways. Among them, the pathway associated with intramuscular fat was the AMPK signaling pathway, and the related genes were *PPARGC1A* and *ADIPOQ*; Immunohistochemical studies showed that PPARGC1A and ADIPOQ proteins were expressed in connective tissues, cell membranes, and, to a lesser extent, the cytoplasm of the longest dorsal muscle of the little-tailed frigid sheep; Real-time PCR and Western Blot validation showed that *PPARGC1A* and *ADIPOQ* were both expressed in the longest dorsal muscle of the little-tailed frigid sheep at different ages, and there were age differences in the amount of expression. The *ADIPOQ* gene was negatively correlated with the intramuscular fat content of the longest dorsal muscle, and the *PPARGC1A* gene was positively correlated with the intramuscular fat content of the longest dorsal muscle; As inferred from the above results, the *ADIPOQ* gene was negatively correlated with the intramuscular fat content of the longest dorsal muscle (*r* = -0.793, *P* < 0.05); and the *PPARGC1A* gene was positively correlated with the intramuscular fat content of the longest dorsal muscle *r* = 0.923, *P* < 0.05).

**Conclusions:**

Based on the above results, it can be inferred that the *ADIPOQ* gene is negatively correlated with the intramuscular fat content of the longest back muscle (*r* = -0.793, *P* < 0.05); the *PPARGC1A* gene is positively correlated with the intramuscular fat content of the longest back muscle (*r* = 0.923, *P* < 0.05).

**Supplementary Information:**

The online version contains supplementary material available at 10.1186/s12864-024-10486-w.

## Introduction

The adipose tissue of livestock and poultry is divided into four types according to the location of fat deposition, i.e., intramuscular fat (IMF), subcutaneous fat (SCF), interstitial fat (IMTG) and visceral adipose tissue (VAT) [1]. The formation of subcutaneous fat deposits and visceral fat deposits in livestock and poultry consumes a lot of energy, so the economic value of subcutaneous fat deposits and visceral fat is low [2–4]. Contrary to subcutaneous and visceral fat, it is the intramuscular and intermuscular fat that play a pivotal role in determining the quality of meat in livestock and poultry. As a result of the pursuit of meat production, livestock and poultry meat production has favored the aspect of improving the growth rate of livestock and increasing the rate of lean meat, which in turn has led to a decline in the quality of meat, directly affecting the content of intramuscular fat. Intramuscular fat content affects the color, tenderness, tethering force, shear force value, flavor, juiciness, and other indicators of the main factors. Intramuscular fat refers to the tissue between the skeletal muscle fibers, that is, the fat entrapped in the membrane of the connective tissue of the muscle, which is one of the important indicators of the quality of meat [5]. Intramuscular fat gives the quality of muscle meat in livestock and poultry. It is generally believed that muscle with an IMF content between 2% and 3% has the best taste and flavor. As with other traits of muscle quality, factors affecting intramuscular fat content include heredity, age, sex, and nutritional level and are regulated by functional genes [6].

The small-tailed han sheep belongs to the Mongolian sheep lineage and is a famous livestock breed in China, which is mainly characterized by roughage tolerance, more stable genetic performance, high fertility, and a high slaughter rate [7, 8]. However, small-tailed han sheep exist; adult small-tailed cold sheep meat color and meat quality are white; taste and flavor are not ideal; and there are other shortcomings [9]. IMF content can improve the taste and flavor of the meat, so improving IMF content in small-tailed cold sheep is one of the main methods to improve the color and quality of meat in small-tailed cold sheep. IMF levels are influenced by a variety of factors, including age, gender, and environment [10]. Based on this, the paper compares the different genes in the longest dorsal muscle of small-tailed cold sheep of different ages, using them as test animals and employing transcriptome sequencing. It determines the intramuscular fat (IMF) content of the longest dorsal muscle, the index of the longest dorsal muscle fibers, and screens the different genes that may affect the deposition of IMF through transcriptome sequencing. The paper also analyzes the expression characteristics of these different genes. This can provide some theoretical references for clarifying the mechanism of differentiation of IMF deposition in small-tailed cold sheep and improving the quality of their meat.

## Materials and methods

### Experimental design and sample collection

The test animals were selected from Guofeng Livestock Breeding Sheep Farm, China. 20 ewes of similar age and weight were selected and kept in the same pen, and the simultaneous estrus technique was used to ensure that the selected ewes were lambed at the same time. Fifteen lambs were selected and each sheep was numbered. Before the experiment, a random number generator was used to randomly assign the sheep to different age groups and the 15 lambs were randomly divided into five groups with three lambs per month (aged 1, 2, 3, 6, and 12 months respectively). All lambs were fed under the same conditions and at the same base level. After testing sheep in each age group, the longest dorsal muscle between the 12th and 13th ribs was collected and placed in liquid nitrogen and 4% formaldehyde fixative in a sterile environment for subsequent testing (A: 1 month old; B: 2 months old; 3: 1 month old; 6: 1 month old; 12: 1 month old; 3 in each group). These test sheep were slaughtered by electroshock unconsciousness, which reduces pain and stress by passing an electric current through the animal to render it rapidly unconscious. Generally, a two-stage low-voltage alternating current is used, the electric shock current is 1 ~ 1,25 A, the voltage is 75 ~ 125 V, and the duration is 3 ~ 10 s. Specific operation: the electric clamps are placed on both sides of the brain of the sheep so that the electric current passes through the brain of the sheep to cause the sheep to be unconscious. In order to improve the effect of knockout before the electric shock, you can cut off the electric shock position of the wool or wet electrodes.

### Determination of muscle fiber parameters and intramuscular fat content

After taking the longest back muscle of sheep, the blood stain was removed by washing with PBS, and then fixed with 4% paraformaldehyde. A 0.5 × 0.5 × 0.5 tissue sample will be cut out, then the sample will be rinsed in tap water for 12 h, and then dehydrated with different gradients of ethanol (30%, 50%, 70%, 80%,95% and 100% alcohol for 2 h each). The tissue was then soaked in xylene (30 min) and treated with low - and high-melting wax. Finally, the sections were embedded in paraffin and sliced by conventional methods. hematoxylin-eosin staining was used.

The tissue sections were stained by staining method and then stained with biological microscope Olympus BX53 (Japan). The diameter of 20 adjacent muscle fibers were measured in each section and their average values were obtained. The muscle fiber density of each section was measured under 3 fields of 2000µm2.

In this experiment, the IMF content in the longest muscles of the back was determined by Soxhlet extraction method according to the National standard of the People’s Republic of China (GB5009.6-2016). The specific step is to use Soxhlet extraction method to extract the target components. First, the fully mixed sample 2 g to 5 g, accurate to 0.001 g, is weighed and all of it is transferred into the filter paper cartridge. Then, the filter paper cartridge is placed in the Soxhlet extractor extractor and connected to the receiving bottle that has been dried to constant weight. Add anhydrous ether or petroleum ether through the upper end of the Soxhlet extractor condensate tube to two-thirds of the volume of the receiving bottle. Then, the device is heated on a water bath, so that the anhydrous ether or petroleum ether is continuously pumped back, the reflux frequency is 6 to 8 times per hour, and the total extraction time is 6 to 10 h. The end of extraction is judged by the use of a frosted glass rod to pick up 1 drop of the extract. If there is no oil spot on the frosted glass rod, the extraction is complete. After extraction is complete, remove the receiving bottle, recover anhydrous ether or petroleum ether, and dry on a water bath when the solvent in the receiving bottle remains 1mL to 2mL. Then, the receiving bottle is dried at 100 ° C ± 5 ° C for 1 h, and then placed in the dryer to cool for 0.5 h before weighing. Repeat drying, cooling, and weighing operations until the receiving bottle is of constant weight (i.e. the difference between two consecutive weights does not exceed 2 mg).

### RNA isolation, library preparation, and sequencing

In this study, RNA was extracted from tissues or cells using standard extraction methods, followed by strict quality control of RNA samples, mainly through Agilent 2100 bioanalyzer (accurate detection of RNA integrity). Novogene Co. Ltd (Tianjin, China) conducted cDNA library preparation and RNA-seq, The NEBNext® Ultra™ II RNA Library Prep Kit for Illumina® (NEB #E7775L) was used. Get clean reads from raw data by removing low-quality reads and reads that contain adapters or poly-N. The Q20, Q30 and GC content of the clean data is then calculated. All downstream analysis is performed using clean, high-quality data [11–14].

### Differentially expressed Gene (DEG) analysis

Genes were compared between samples using the DESeq method using the DESeq2 (Anders et al., 2014) software and log2 (fold change, FC) ≥ 1 with a *P* - value < 0.05 as a criterion for screening DEGs. GO and KEGG functional annotation of DEGs was performed by Nohe Cloud Platform (https://magic.novogene.com/customer/main#/small-tools/1).

### Functional enrichment analysis

To identify functions and pathways associated with the DEGs, Gene Ontology (GO) and Kyoto Encyclopedia of Genes and Genomes (KEGG) pathway enrichment analyses were performed using the NovoMagic CloudPlatform (https://magic.novogene.com/customer/main#/loginNew). A cut-off of *P* < 0.05 was used to screen significant functions and pathways. We further screened core pathways and candidate genes by consulting the published literature.

### Validation of DEGs using qRT-PCR

In this experiment, the expression of muscle fat-associated DEGs in sheep was detected by qRT-PCR (Bio-Rad CFX96, USA) RNA was extracted using a polysaccharide polyphenol total RNA kit(TRIzol extraction method) (Tian gen, Beijing, China).The quality of RNA was assessed by agarose gel electrophoresis in this experiment. Cycling conditions were as follows: pre-denaturation at 95 °C for 30 s, followed by 40 cycles of denaturation at 95 °C for 5 s and re-denaturation at 60 °C for 10 s, and finally 72 °C for 15 s. Each sample was tested 3 times. Normalization was performed using the β-actin gene as an internal control. Data were analyzed using spss22 software and relative expression was calculated using the 2^−ΔΔCt^ method. Primers were designed using NCBI online (https://www.ncbi.nlm.nih.gov/tools/primer-blast/) [15] and are listed in Table 1.

**Table 1:** Primer sequences for fluorescent quantitative PCR

Genes	Sequence of primer	Size(bp)
*PPARGC1A*	F : GCAGAGAGTATGAGAAGCGGGAATC	131
	R : CCTCAGTTCTGTCCGTGTTGTGTC	
*DIPOQ*	F : ACAGGTTGGATGGCAGGCATTC	143
	R : CCAGTTTCACCAGTGTCACCCTTAG	
*STAT5A*	F : GCGGAAGCAGCAGACCATCATC	126
	R : GCCAACTTCTCACACCAGGACTG	
*PPP1R3A*	F : TGGCTCGGAGAGTGACACCTTC	98
	R : CGTTAGCATGGTAGGCGACACAG	
*Myf6*	F : TTAGAAGTGGCAGAGGGCTCTCC	112
	R : CATGTTCCTCTCCGCTGCTGTC	
*β-actin*	F : CCATCGGCAATGAGCGGTTCC	146
	R : CGTGTTGGCGTAGAGGTCCTTG	

### Western blotting analysis

Expression of PPARGC1A and ADIPOQ-associated proteins in intramuscular fat samples from the most extended dorsal muscles between groups was quantified using protein blotting. Electrophoresis gel electrophoresis was performed on denatured proteins. For each sample, a volume containing 20 µg of total protein was transferred to 4–20% SDS-PAGE and Tris-Glycine SDS electrophoresis buffer for 1 h. The transferred proteins were bound to the surface of the PVDF membrane for 22 min and 30 min, respectively. The membranes were closed with 5% skim milk powder for 2 h at room temperature and then incubated with primary antibody (rabbit IgG, Thermo Fisher Scientific, China) for 12 h at 4℃. Finally, the membrane was incubated with goat anti-rabbit IgG (Rabbit IgG, Thermo Fisher Scientific, China) for 2 h at room temperature. The membranes were then visualized.

### Statistics analysis

Statistical analysis Statistical indicators were expressed as the mean of 3 replications. One-way ANOVA with Duncan’s multiple-range test was used. Significant differences were indicated using different letters (*P* < 0.05). Statistical analysis was performed using SPSS22 and plotted using Origin 2022.

## Results

### Measurement of muscle fiber parameters and intramuscular fat in the longest dorsal muscle of sheep

**Fig. 1:**
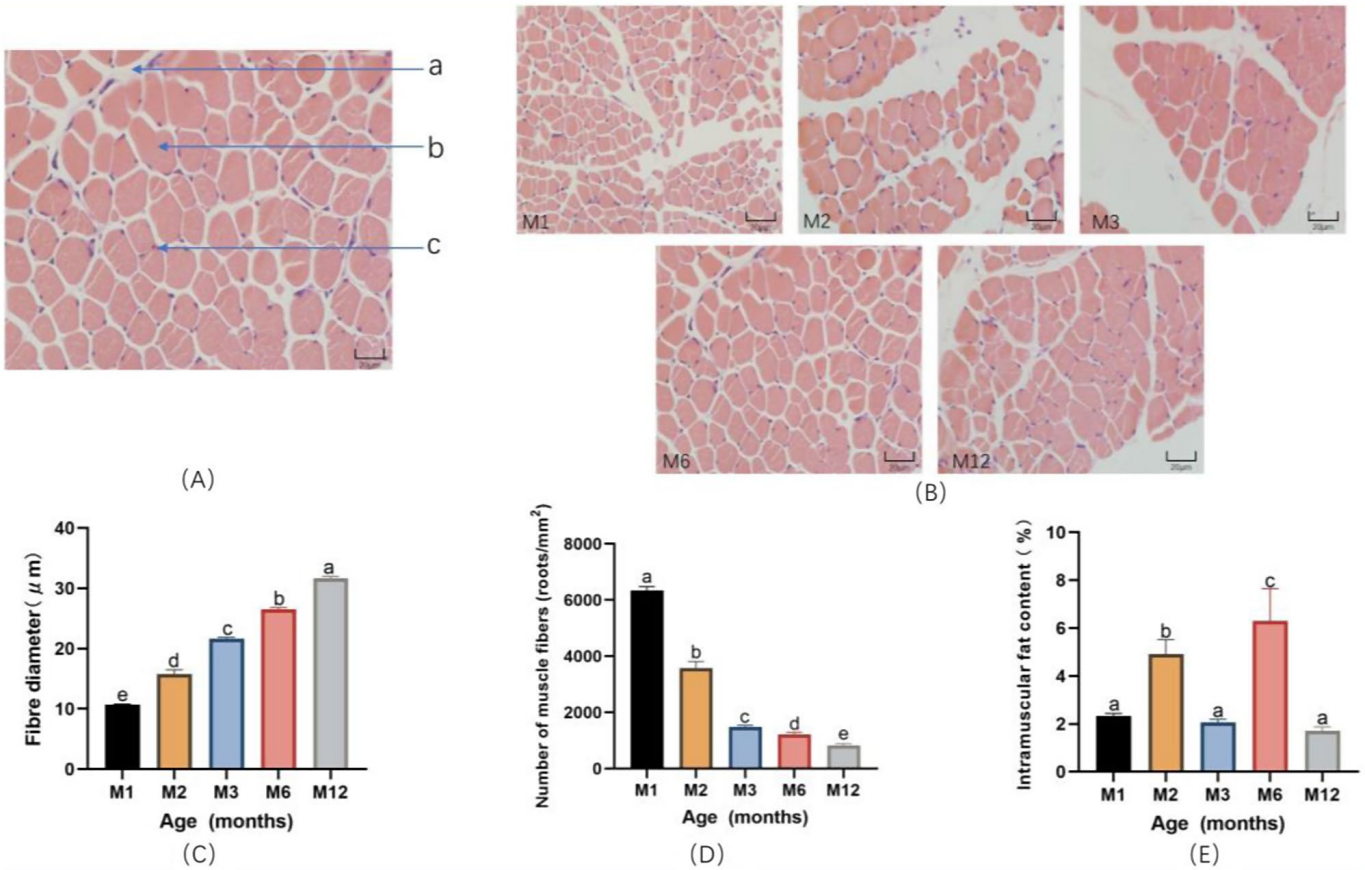
Muscle fiber parameters and intramuscular fat content of the longest dorsal muscle of sheep of different ages. (**A**) Schematic diagram of the muscle fiber structure of the longest dorsal muscle of sheep, Where “a” is connective tissue, “b” is muscle fiber, and “c” is cell nucleus. (**B**) HE staining of the longest dorsal muscle of sheep at different months of age. (**C**) Diameter of muscle fibers of the longest dorsal muscle of sheep at different months of age. (**D**) Density of muscle fibers in the longest dorsal muscle of sheep at different months of age. (**E**) Intramuscular fat content of the longest dorsal muscle of sheep at different months of age

Microscopic observation of the most extended dorsal muscle section of small-tailed cold sheep, the longest dorsal muscle fiber diameter, and myofiber density of different months of age sheep were compared, as Fig. 1A shows the most extended dorsal muscle tissue of 6-month-old sheep, respectively, connective tissues, and myofibrils and nuclei.

In this experiment, after HE staining the muscle fibers of the longest dorsal muscle of sheep at different monthly ages, the muscle fibers showed pink color, and the nuclei of myocytes were blue in the tissue sections. As can be seen from Fig. 1B, under the microscope 400× field of view, the area of the longest dorsal muscle fibers of sheep gradually increased with the increase of the age of the month, the number of muscle fibers in each field of view gradually decreased, and the diameter of the muscle fibers gradually increased.

As can be seen from Fig. 1C, the muscle fiber diameter of the longest dorsal muscle of sheep increased significantly (*P* < 0.05) with the increase of the month’s age. The muscle fiber diameter of the longest dorsal muscle of 1-month-old sheep was significantly lower (*P* < 0.05) than that of the four periods (10.67 ± 0.13), and the diameter of the longest dorsal muscle of 12-month-old sheep was significantly higher (*P* < 0.05) than that of the four periods (31.64 ± 0.33).

As can be seen from Fig. 1D, the number of muscle fibers (roots/mm^2^) of the longest dorsal muscle of sheep decreased significantly (*P* < 0.05) with the increase of age in months, the number of muscle fibers of the longest dorsal muscle of 1-month-old sheep was significantly higher (*P* < 0.05) than that of the four periods (*P* < 0.05) at the age of 1-month; the number of muscle fibers of the longest dorsal muscle of sheep at the age of 12-months was significantly lower (*P* < 0.05) than the four periods (*P* < 0.05) at the age of 1-month to 12-months (*P* < 0.05). ); the decreasing trend of the number of muscle fibers from 1 month old to 2 months old was more significant; the decreasing trend of the number of muscle fibers from 3 months old to 12 months old was more minor.

In this experiment, the intramuscular fat content of the longest dorsal muscle of sheep at different ages was compared, and the experiment results can be seen in Fig. 1E. The intramuscular fat of the longest dorsal muscle of sheep at the age of 6 months was significantly higher than that at the other 4 periods (*P* < 0.05). The intramuscular fat of sheep’s longest dorsal muscle at 12 months was significantly lower than that at the age of 2 months and 6 months (*P* < 0.05). The overall trend of intramuscular fat in the longest dorsal muscle of sheep showed an “*M*” shape, with a significant increase in intramuscular fat content from 1 month to 2 months of age (*P* < 0.05); a significant decrease in intramuscular fat content from 2 months to 3 months of age (*P* < 0.05); a significant increase in intramuscular fat content from 3 months to 6 months of age (*P* < 0.05); a significant increase in intramuscular fat content from 6 months to 12 months of age (*P* < 0.05); and a significant increase in intramuscular fat content from 6 months to 6 months of age (*P* < 0.05). Intramuscular fat content decreased significantly (*P* < 0.05) from 6 months of age to 12 months of age.

### Statistics and evaluation of sequencing data

According to Tables 2 and 110.96 G of data were obtained for the 12 samples, with an average valid data for a single sample of 7.40 G. The Q30 (percentage of total bases with a Phred value greater than 30) were all above 90%. These data suggest that transcriptome sequencing can be used for further testing.

**Table 2:** Statistics of the quality and alignment efficiency of transcriptome sequencing

Sample	Raw Reads	Clean Reads	Clean Base	Q30 (%)	GC Contents (%)
A1	61,226,226	59,305,782	8.9G	92.63	51.8
A2	63,575,902	60,940,492	9.14G	92.89	51.38
A3	46,746,846	44,970,186	6.75G	92.81	51.89
B1	46,185,792	43,500,834	6.53G	92.54	49.87
B2	60,898,672	58,519,554	8.78G	94.05	50.24
B3	55,110,478	51,792,926	7.77G	92.74	51.66
C1	46,043,166	44,981,190	6.75G	92.94	52.14
C2	47,074,300	46,054,732	6.91G	92.18	52.31
C3	49,430,414	48,178,170	7.23G	92.9	52.08
D1	44,271,678	43,187,532	6.48G	92.37	51.88
D2	59,321,052	57,926,764	8.69G	92.59	51.65
D3	46,024,440	44,845,536	6.73G	92.15	51.76
E1	48,282,654	47,186,204	7.08G	92.55	52.4
E2	50,464,458	48,865,448	7.33G	92.9	51.53
E3	40,506,592	39,256,230	5.89G	92.45	51.47

### DEGs

In order to screen for candidate genes significantly associated with changes in intramuscular fat in the longest dorsal muscle of sheep at different months of age, this experiment was conducted using 1 month of age as a control group, which was compared with other age groups, respectively. DESeq2 was used to obtain DEGs between different combinations.

**Fig. 2:**
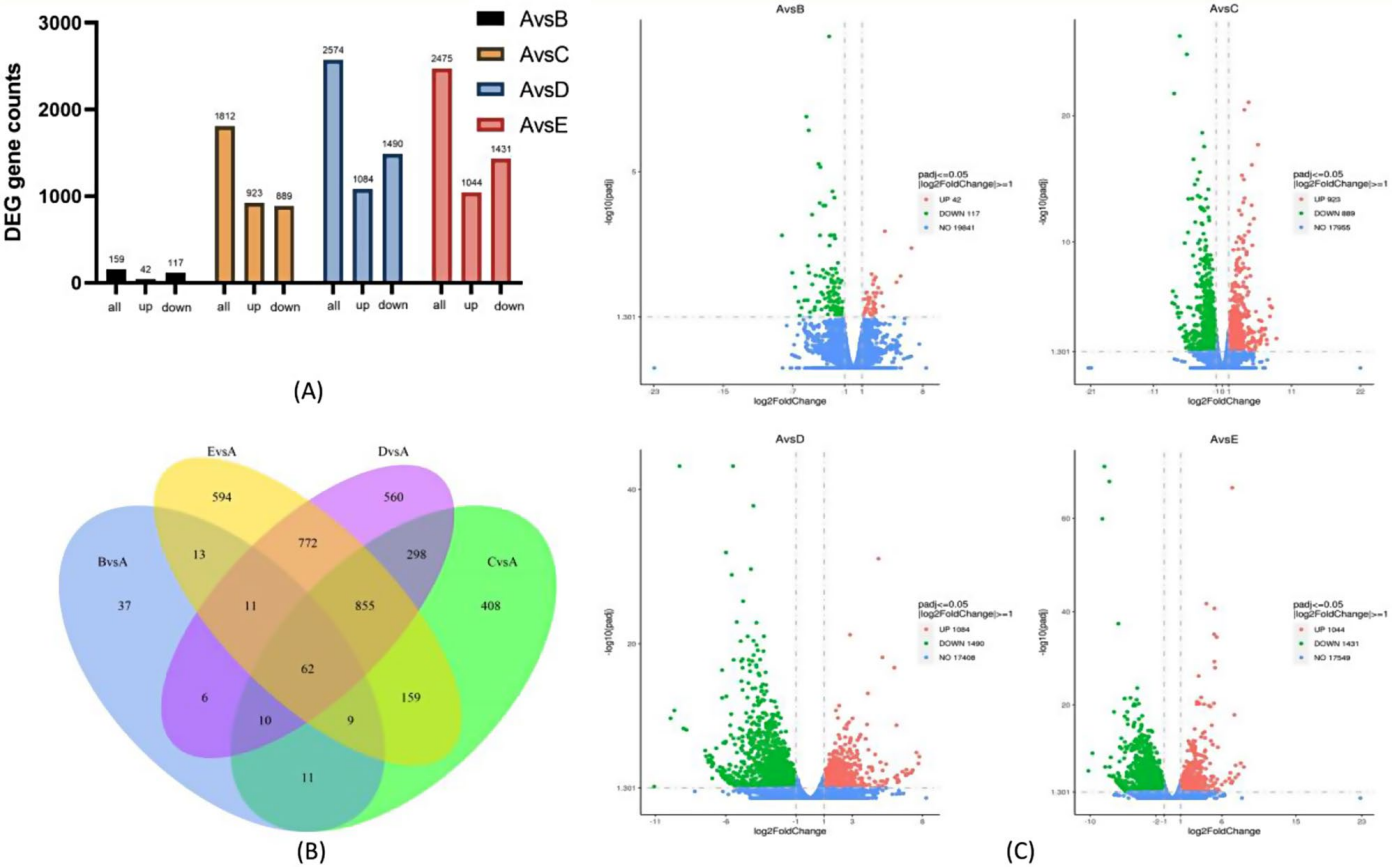
Differentially expressed genes (DEG) between different months of age using 1 month of age as control. (**A**) Number of differentially differentiated genes at different months of age using 1 month of age as a control; (**B**) Venn diagram of the comparison of DEGs in the four groups of samples; (**C**) Volcano plot of DEGs

Using 1 month of age as the control group, 20,000, 19,767, 19,982, and 20,024 genes were identified in each of the four groups at 2 months of age, 3 months of age, 6 months of age, and 12 months of age. Using *P* < 0.05 and log_2_|Fold-Change|>1 as thresholds, 159 (42 up-regulated and 117 down-regulated genes), 1812 (923 up-regulated and 889 down-regulated genes), 2574 (1084 up-regulated and 1490 down-regulated genes) and 2475 (1044 up-regulated and 1431 down-regulated genes) differential genes, for a total of 62 genes for all combinations(Figure 2).

### Functional enrichment analysis and KEGG analysis of the DEGs

**Fig. 3:**
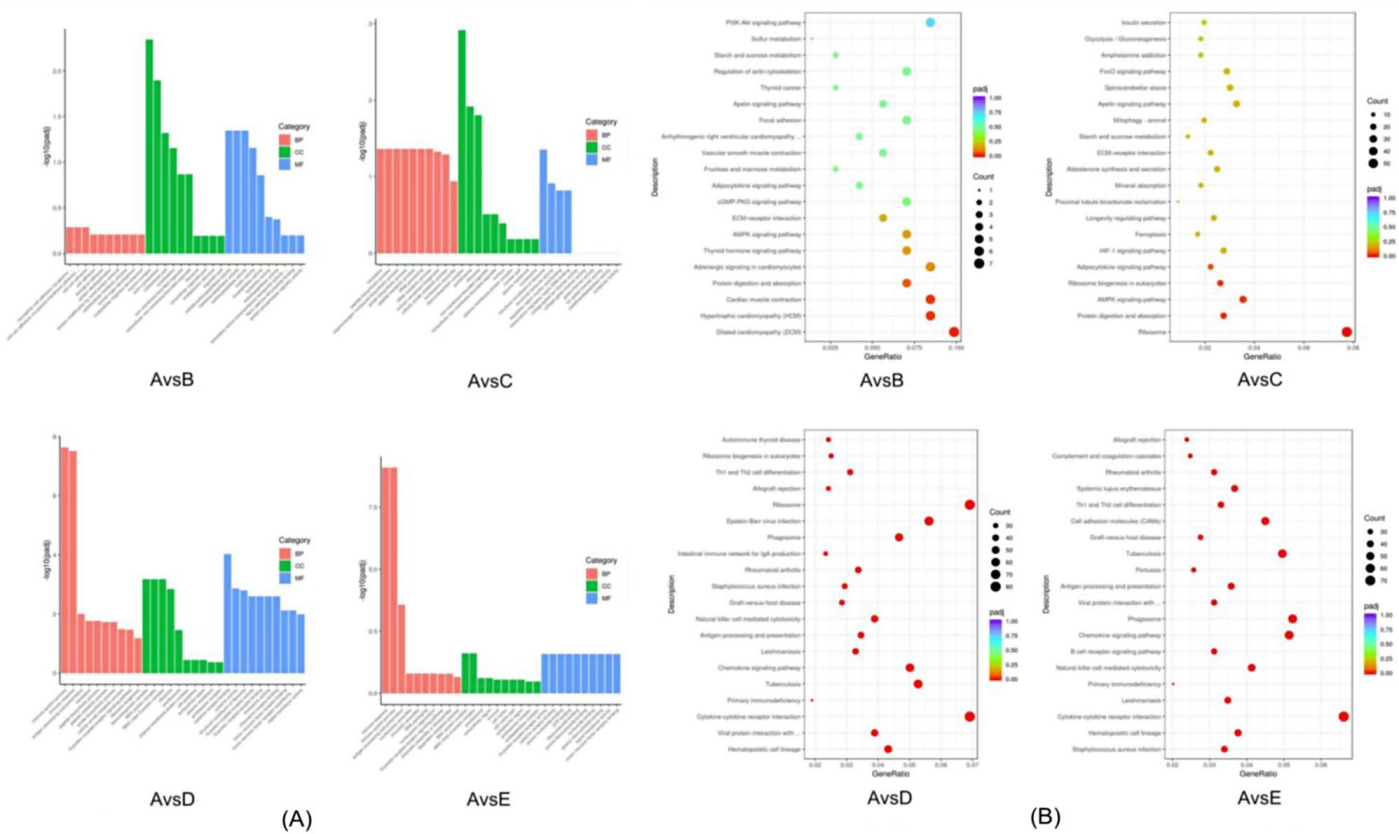
GO enrichment and KEGG enrichment of differential genes between different combinations (**A**) Histogram of GO enrichment analysis of DEGs in different combinations. (**B**) Scatter plot of KEGG enrichment in different combinations

Next, GO functional enrichment analysis was performed to explore the functions of the DEGs. GO terms included three categories: cellular composition, molecular function, and biological processes. Using 1 month of age as a control, each group had 6, 12, 30, and 22 significantly enriched GO entries. The top 30 GO entries between different age groups were filtered by *P*-value ordering (the most significant 30 Terms were selected to be plotted in a bar chart for presentation, or all Terms if there were less than 30). As shown in Fig. 3A, GO entries between different age groups were both common and unique to their age groups (Fig. 3A).

KEGG enrichment of differential genes allowed the prediction of the relevant pathways regulated by the genes of interest. According to the differential gene screening criteria, four age groups were formed using 1 month old as the control group with 2 months old, 3 months old, 6 months old and 12 months old. Pathway enrichment analysis of differentially expressed genes by KEGG was performed in the 2 months, 3 months, 6 months, and 12 months groups, and 12, 32, 61, and 60 significantly enriched pathways were identified using *P* < 0.05 as the condition of significant enrichment, respectively.

Figure 3B (the 20 most significant KEGG pathways were selected and plotted as scatter plots; if less than 20, then all pathways were plotted) shows that most of the pathways were disease-related pathways, except for the AMPK signaling pathway, which was screened for its relevance to intramuscular adiposity in all months of age, which existed in all combinations of months of age. The AMPK pathway is differentially expressed in different combinations at different ages, with the genes associated with intramuscular fat deposition being *PPARGC1A* (peroxisome proliferator-activated receptor coactivator) and *ADIPOQ* (adiponectin).

### Transcriptomics qRT-PCR validation

**Fig. 4:**
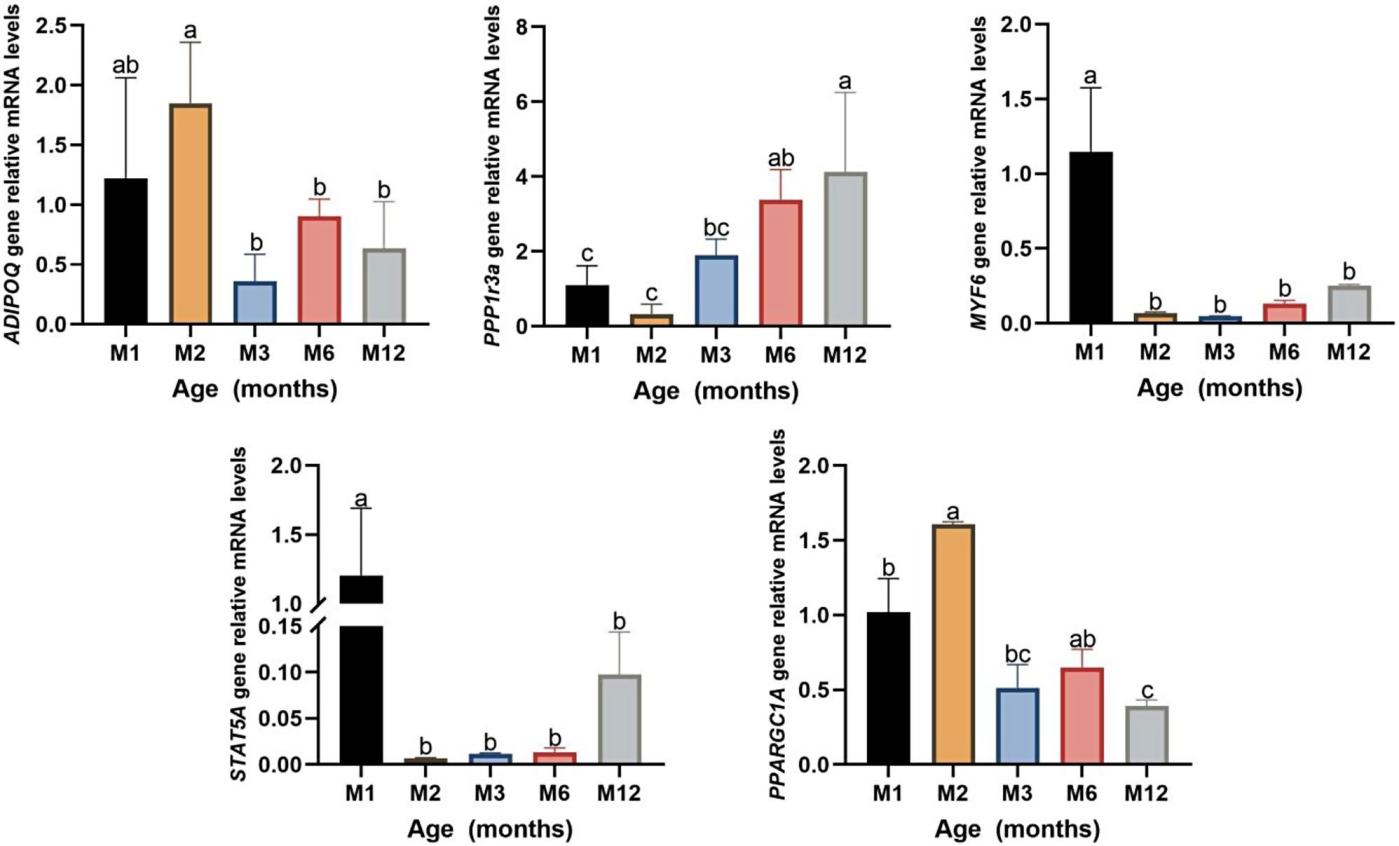
The mRNA relative expression level of DEG was verified by qRT-PCR

Gene expression validation: This experiment selected five DEGs, including *PPARGC1A*, *DIPOQ*, *STAT5A*, *PPP1R3A*, and *Myf6*. qRT-PCR results confirmed that the expression patterns of these genes were consistent with RNA-seq (Fig. 4). These results confirmed the high confidence in the gene expression results of blood transcriptome sequencing analysis.

### Immunohistochemistry and Western blotting test results

**Fig. 5:**
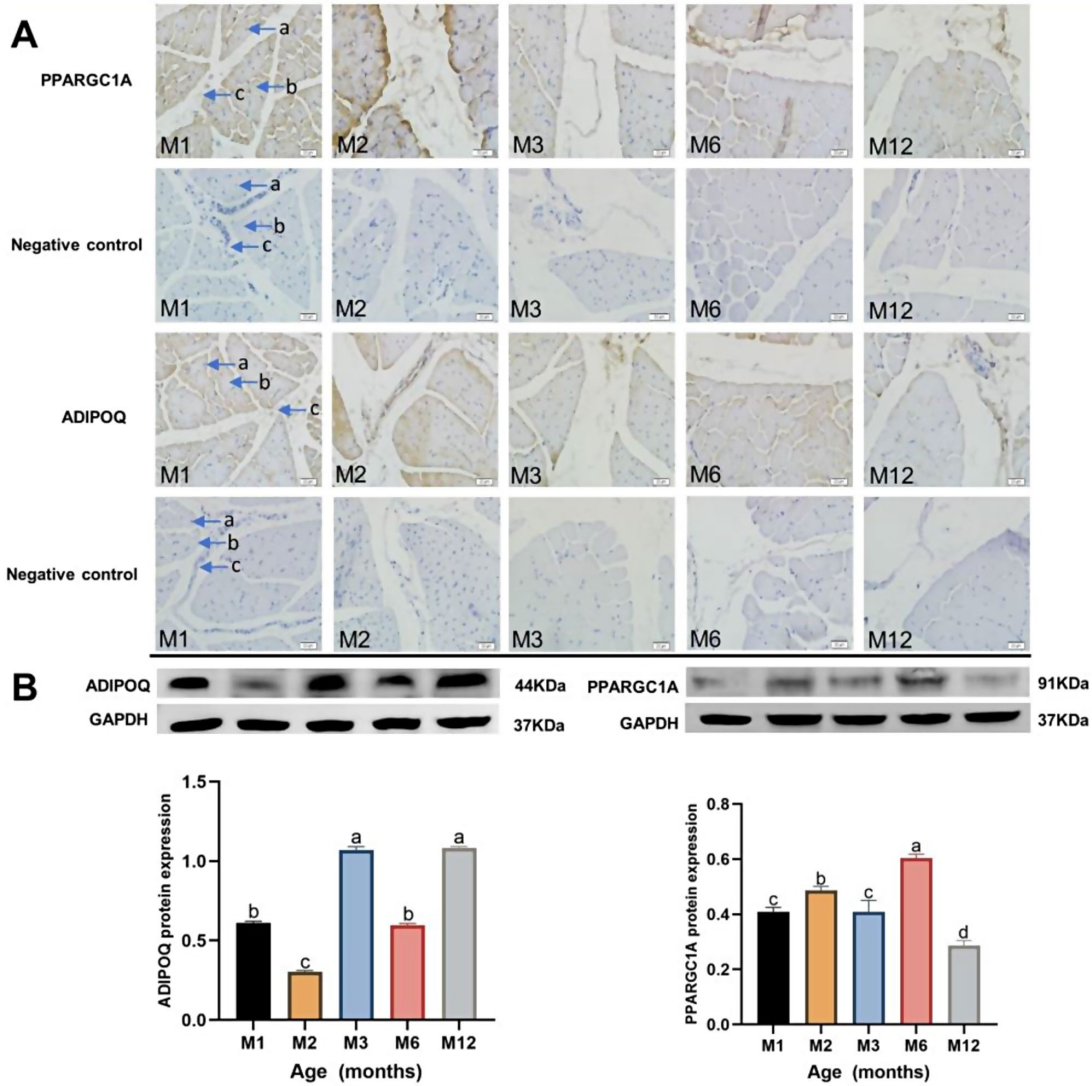
Immunohistochemistry and Western blotting test results. (**A**) Immunohistochemical results of PPARGC1A and ADIPOQ proteins in the intramuscular fat of the longissimus dorsi muscle at different ages. The “a” is nucleus, “b” is cytoplasm and “c” is connective tissue. (**B**) PPARGC1A and ADIPOQ Western blotting of intramuscular fat in the longest dorsal muscle of sheep of different months of age using 1 month of age as control

The immunohistochemistry technique was used to study the localization of PPARGC1A protein and ADIPOQ protein expression in the longest dorsal muscle of sheep at different months of age. The results of Fig. 5(A) showed that PPARGC1A protein and ADIPOQ protein were expressed in the longest dorsal muscle of sheep at different months of age and that both PPARGC1A protein and ADIPOQ protein were positively expressed in the cytoplasm and connective tissue.

The protein expression of PPARGC1A and ADIPOQ in the longest dorsal muscle of sheep at different periods was determined by protein immunoblotting. The gray values detected by immunoblotting were processed and analyzed. The obtained data were normalized, and the relative expression of the protein levels of the longest dorsal muscle of sheep, PPARGC1A and ADIPOQ, are shown in Fig. 5 (B). The protein expression of ADIPOQ was the maximum of 5 periods at 12 months of age and the minimum of 5 periods at 2 months of age. The expression of ADIPOQ protein in the longest muscle of the back of sheep was the maximum value in five periods at 12 months of age, and the minimum value in five periods was at 2 months of age. Taking 1 month of age as the control group, the expression of ADIPOQ protein in the longest muscle of sheep at 1 month of age was significantly higher than that in the longest muscle of sheep at 2 months of age (*P* < 0.05), the expression of ADIPOQ protein in the longest muscle of sheep at 1 month of age was significantly lower than that in the longest muscle of sheep at 3 months of age and 12 months of age (*P* < 0.05). ADIPOQ protein expression was negatively correlated with the intramuscular fat content of the longest dorsal muscle of sheep at different ages (*r*=-0.793, *P* < 0.05). The PPARGC1A protein expression in the longest dorsal muscle of sheep was the maximum in five periods at 6 months of age and the minimum in five periods at 12 months of age. The expression of PPARGC1A protein at 1 month of age was significantly higher than that at 12 months of age (*P*<0.05) and significantly lower than that at 2 months of age and 6 months of age, and the overall expression trend of the five periods was “*M*”-shape trend. PPARGC1A protein expression was positively correlated with the intramuscular fat content of the longest dorsal muscle of sheep at different ages (*r* = 0.923, *P* < 0.05).

## Discussion

Lamb meat is rich in high-quality proteins and minerals (e.g., iron and zinc) and has a delicate texture, which is popular among consumers. Myofibers are the most essential component of muscle tissue. The histological properties of myofibers in sheep meat are closely related to their quality, and the diameter and density of myofibers directly impact muscle quality. The most severe impact is the yield of sheep meat [16, 17]. The main factors affecting the quality of sheep meat are pH, meat color, tenderness, intramuscular fat, WHC (WHC refers to the exposure of livestock and poultry meat to external forces such as pressure, grinding, heating, freezing, and thawing), water-holding capacity (which refers to the water that is not easily flowable, accounting for about 80% of all water), and drip loss [18, 19], of which the intramuscular fat, also known as the marbled adipose tissue, is located in the myofibrils and the muscle surroundings next to the connective tissues, and consists mainly of triacylglycerol (TG) and phospholipids, the content of which affects the tenderness, juiciness, and texture of meat. Low levels of intramuscular fat result in dry meat [20, 21]. The transcriptome is all the gene transcripts or RNA species transcribed in a particular cell type, tissue, or organism. It consists of coding RNAs, translated into proteins, and non-coding RNAs, involved in post-transcriptional regulation and thus influence gene expression. Transcriptome research aims to explain key aspects of genomic functional outcomes by comparing changes in gene expression in cells or tissues under specific conditions or disease states [22–24].

After an animal is born, muscle growth depends on the increase in the diameter of muscle fibers, and its number does not change [25]. Muscle fiber diameter, density, muscle fiber type, water, protein, and fat content in the muscle all affect meat quality. The smaller the diameter and the higher the density of muscle fibers, the higher the fat content in the muscle, the higher the tenderness, and the better the meat quality [26]. The basic unit of muscle tissue is the muscle fiber, which forms the muscle bundles, and the size of the bundles directly affects the tenderness and shear force of livestock meat and affects the flavor of its livestock meat [27, 28]. In this study, the dorsal longest muscle fiber diameter and the number of muscle fibers were analyzed in sheep of different months of age, and the results showed that the diameter of the dorsal longest muscle fibers increased significantly with the increase of months of age (*P* < 0.05). The results of this test followed approximately the same trend as those of other testers [29–31]. As sheep grow, muscle fiber density and tenderness decrease, and this paper found significant differences in muscle fiber density at different growth stages. Therefore, controlling the slaughter month age can increase their sheep’s economic value. Backfat thickness, intramuscular fat content, eye muscle area, tenderness, and meat color are indicators for evaluating meat quality traits in livestock and poultry in the conventional sense, of which intramuscular fat has become one of the essential assessment criteria [32]. Intramuscular fat content affects sheep’s tenderness, meat color, and flavor indicators [33]. The results showed that the intramuscular fat content fluctuated in an “*M*” shape with age, reaching the maximum value in five periods at 6 months of age and the minimum value in five periods at 12 months of age. Some experimentalists determined the intramuscular fat content of the longest dorsal muscle of 2, 21, 56, 84, and 112 days Euler rams, and the intramuscular fat content of the longest dorsal muscle of Euler rams increased gradually with the increase of age [34]. The fat content of the longest dorsal muscle of the sheep was consistent with the trend from 1 month to 2 months of age in this paper. Lin Y et al. [35] measured the fat content of the longest dorsal muscle of the sheep at 2 and 9 months of age, and the results of the test showed that the fat content of the longest dorsal muscle of the sheep increased with the increase of the age of the month. The results showed that the fat content of the sheep increased gradually with the month’s age. The main reason for the increase in muscle fiber diameter with age in sheep is that satellite cell activity, hormone regulation and adequate nutrition promote muscle hypertrophy. As sheep age, the increase in intramuscular fat content is driven by metabolic changes, hormonal changes, genetic predisposition, nutritional factors, and external environment. These changes improve meat quality by improving flavour, tenderness and juiciness, and understanding these physiological and metabolic factors is critical to optimizing breeding, feeding and management practices for optimal sheep meat quality. In this experiment, the intramuscular fat content decreased at 2 months of age and 3 months of age, which may be attributed to the fact that 2 months of age and 3 months of age are the weaning period of lambs, which affects the intramuscular fat content of the longest dorsal muscle [36]. The intramuscular fat content of the longest dorsal muscle in this experiment was significantly lower at 12 months than at 6 months. The probable reason for this may be the predominance of myofiber development in the muscle at 12 months of age, as evidenced by the evidence provided in the previous parameters for myofibers. The other reason could be seasonal, as the sheep in this trial were slaughtered at 12 months of age during the winter season, which, in combination with the location of the present trial, is hypothesized to be due to a decrease in intramuscular fat content due to cold.

Intramuscular fat is a complex trait that is affected by various factors, such as nutrition, type, and deposition time, and it is involved in regulating a variety of biological processes, such as hormones, muscle development, and fat deposition. In sheep farming, intramuscular fat content is essential in producing high-quality lamb meat. In this chapter, using small-tailed cold sheep as test animals, we screened the high expression genes and differential genes related to the longest dorsal muscle at 1 month of age versus 2, 3, 6, and 12 months of age and screened candidate genes that form the differences in intramuscular fat through transcriptome sequencing and analysis. Intramuscular fat is one of the main factors affecting the quality of livestock and poultry meat, and in this chapter, two candidate genes related to intramuscular fat deposition, *PPARGC1A* ((peroxisome proliferator-activated receptor coactivator) and *ADIPOQ* (lipocalin), were identified through differential gene screening.

*PPARGC1A* (peroxisome proliferator-activated receptor coactivator) plays a crucial role in several aspects of glucose, lipid, and energy metabolism, and this gene is involved in coordinating metabolic processes in the liver, adipose tissue, and muscle [37]. Lipocalin (*ADIPOQ*) is a biologically active adipokine secreted by adipose tissue. It plays a vital role in regulating fat metabolism, body energy homeostasis, glucose, and body energy homeostasis [38]. Some foreign scholars proved that the *PPARGC1A* gene plays a vital role in energy and fat metabolism in pigs by measuring the relationship between the expression levels of the longest dorsal muscle and backfat and the expression levels of downstream-related genes [39]. The *ADIPOQ* gene produces Lipocalin. In sheep, the lipocalin gene is located on chromosome 1 and contains three exons and two introns. It exerts its physiological effects mainly by binding to the receptors *ADIPOR-1* and *ADIPOR-2*. It has been shown that the *ADIPOQ* gene can reduce fat by promoting fatty acid oxidation and inhibiting lipid synthesis [40]. It has also been found that the *ADIPOQ* gene is associated with diseases such as diabetes [41], hypertension [42], and neoplasms [43].*ADIPOQ* is mainly secreted by adipose tissues and transported to other body organs through its blood circulation [44].

There are multiple fat cell distributions in the body of livestock and poultry, of which the fat in the subcutaneous, intermuscular, visceral, and mesenteric connective tissues is of low economic value. In contrast, intramuscular fat is the fat in skeletal muscle fibers, improving meat quality and flavor [2, 3, 45]. Intramuscular fat content directly affects meat’s tenderness, flavor, and juiciness. In this chapter, we investigated the protein localization and gene expression of *PPARGC1A* and *ADIPOQ* genes in the intramuscular fat of the longest dorsal muscle of sheep at different ages.

It can be observed by immunohistochemistry that ADIPOQ and PPARGC1A proteins were expressed in sheep dorsal longest muscle intramuscular fat at different stages of growth in sheep dorsal longest muscle junctional tissues, cell membranes, and a small amount of cytoplasm. The mRNA expression and protein expression of *ADIPOQ* and *PPARGC1A* were detected by fluorescence quantitative PCR and protein immunoblotting. The results showed that the mRNA expression and protein expression of ADIPOQ and PPARGC1A were different at different periods. The mRNA expression of the *ADIPOQ* gene at the age of 1 month differed from that at the other 4 months. The 1-month-old ADIPOQ protein expression was significantly lower than that at 3 and 12 months of age and significantly higher than at 2 months of age (*P* < 0.05). mRNA expression of the *PPARGC1A* gene at 1 month of age was significantly different from that at 2 and 12 months of age (*P* < 0.05), and protein expression of PPARGC1A at 1 month of age was significantly different from that at 2, 6, and 12 months of age (*P* < 0.05). Some researchers used the intramuscular fat of the pectoral muscle of Iberian pigs as the research object. Through transcriptome sequencing of the high-fat and low-fat groups, it was found that the expression of the *ADIPOQ* gene in the low-fat group was lower than that in the high-fat group [46]. Some researchers also hypothesized that *ADIPOQ*, *PPARG*, *LIPE*, and other genes might be candidate genes affecting intramuscular fat content by determining the intramuscular fat content of the longest dorsal muscle of purebred Duroc as well as by transcriptome sequencing of the longest dorsal muscle of Duroc [47]. The results of the experiment were the same as the present experiment. However, the expression of *ADIPOQ* in the present experiment was significantly lower at 3 months of age than at 2 months of age, and based on this paper, the variability may be due to differences between varieties and environmental differences.

The mRNA expression of the ADIPOQ gene in this test was inconsistent with the protein amount. Presumably, the gene expression is divided into two levels of transcription and translation, and there is a spatiotemporal interval between the time and site where transcription and translation of eukaryotic gene expression occurs. The results showed that the ADIPOQ gene was negatively correlated with the intramuscular fat content of the longest dorsal muscle, and the PPARGC1A gene was positively correlated with the intramuscular fat content of the longest dorsal muscle.

## Conclusion

This study thoroughly investigated the growth and development of the little-tailed frigid sheep’s longest dorsal muscle. The results showed that the diameter of the longest dorsal muscle of little-tailed frigid sheep increased with the increase of monthly age. In contrast, the number of myofibers decreased with the increase in monthly age. In addition, the intramuscular fat content of the longest dorsal muscle of little-tailed frigid sheep fluctuated with the month’s age. By analyzing the transcriptome sequencing results, we found that the differential genes in the longest dorsal muscle of small-tailed frigid sheep at different months of age were significantly enriched in the GO entry and KEGG pathways. These differential genes were significantly enriched in the AMPK signaling pathway associated with intramuscular fat deposition, which may be an essential factor influencing the intramuscular fat content of the longest dorsal muscle of the little-tailed chilly sheep. It was found that the essential regulatory genes *PPARGC1A* and *ADIPOQ* were both expressed in the longest dorsal muscle of the little-tailed frigid sheep. The expression of these two genes was significantly different in the longest dorsal muscle of the little-tailed chilly sheep at different months of age, which may be an essential factor affecting the diameter of muscle fibers and the number of muscle fibers in the longest dorsal muscle of the little-tailed chilly sheep. However, the intramuscular fat content of sheep is also affected by differences in farming methods, environmental conditions or genetic factors in different regions or farms, which may have different effects on intramuscular fat content. Overall, the present study revealed some crucial mechanisms during the growth and development of the longest dorsal muscle of the kid, which provides an essential theoretical basis for further research and improvement of muscle quality in kids. Future studies can further explore the specific roles of these differential genes and vital regulatory genes in the growth and development of the longest dorsal muscle of the little-tailed frigid sheep to find an effective strategy to improve the muscle quality of the little-tailed frigid sheep.

### Electronic supplementary material

Below is the link to the electronic supplementary material.

Supplementary Material 1

Supplementary Material 2

## Data Availability

Sequence data that support the findings of this study have been deposited in the NCBI with the primary accession code PRJNA1081634.
