# Supplementary material for: Preliminary studies on the molecular mechanism of intramuscular fat deposition in the longest dorsal muscle of sheep
Source: BMC Genomics. 2024 Jun 12;25:592. doi: 10.1186/s12864-024-10486-w (PMC11167792; doi:10.1186/s12864-024-10486-w)
Supplement: Supplementary file 1 — Supplementary Material 1 [file 12864_2024_10486_MOESM1_ESM.docx]

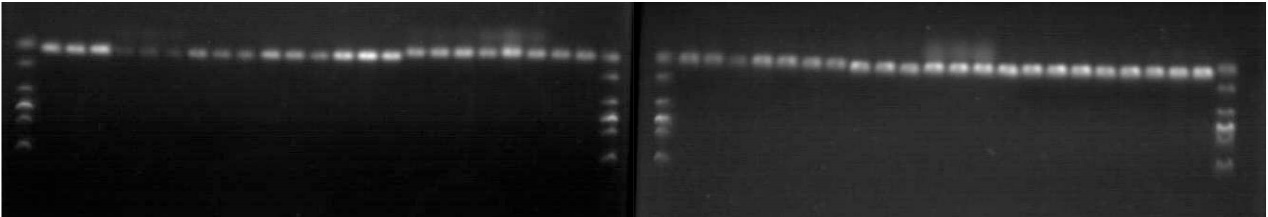


PPARGC1A, DIPOQ, STAT5A, PPP1R3A, Myf6 cDNA agarose gel electrophoresis

Note: From left to right, STAT5A, PPP1R3A, PPARGC1A, Myf6, and DIPOQ genes, with 3 replicates in 5 periods for each 1 gene.
