# Supplementary material for: Preliminary studies on the molecular mechanism of intramuscular fat deposition in the longest dorsal muscle of sheep
Source: BMC Genomics. 2024 Jun 12;25:592. doi: 10.1186/s12864-024-10486-w (PMC11167792; doi:10.1186/s12864-024-10486-w)
Supplement: Supplementary file 2 — Supplementary Material 2 [file 12864_2024_10486_MOESM2_ESM.docx]

1. PPARGC1A protein


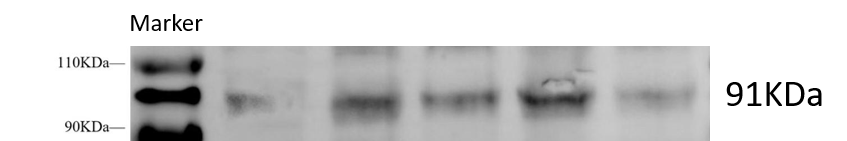


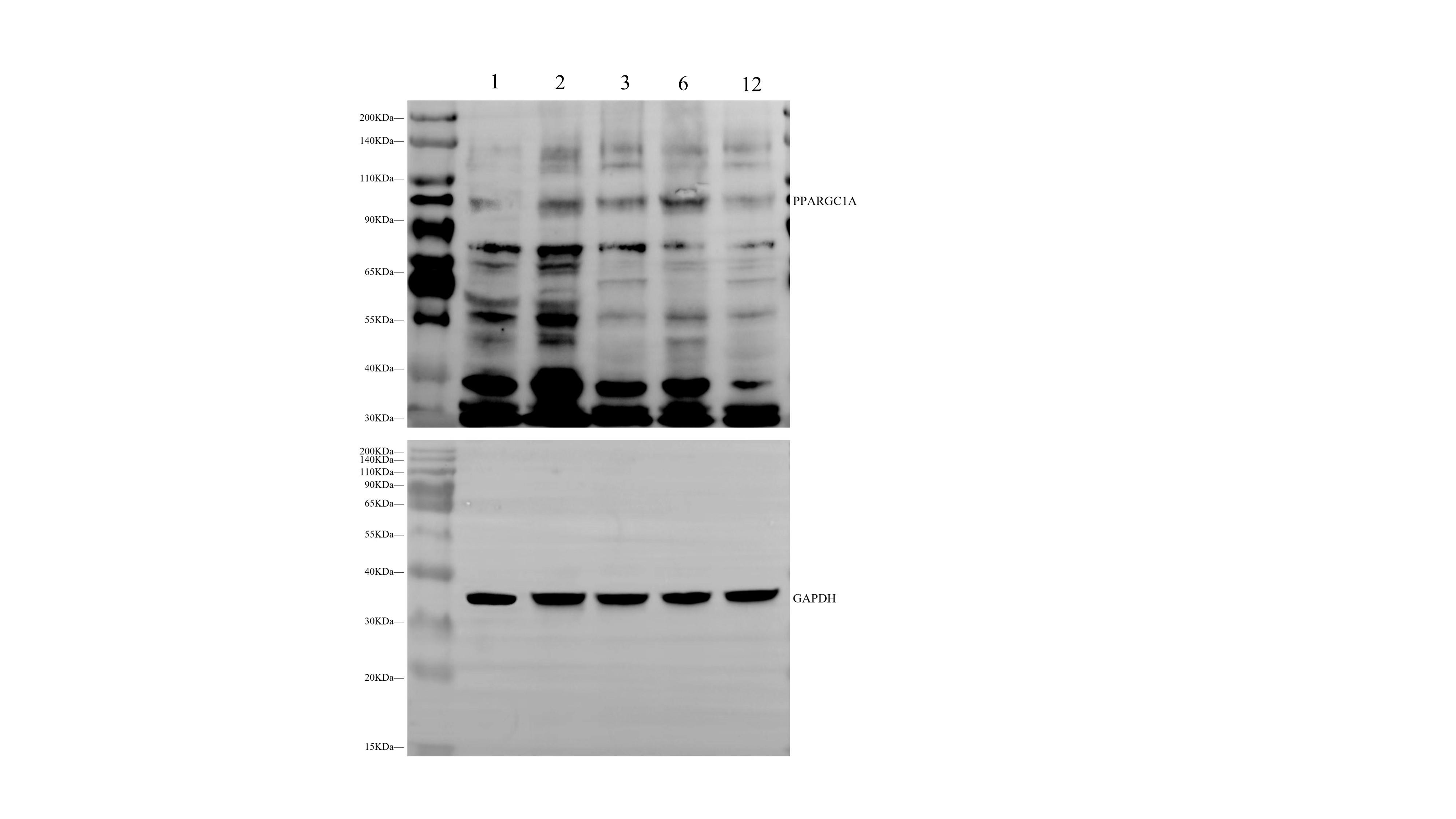


This image is a Western Blot image of the PPARGC1A protein.


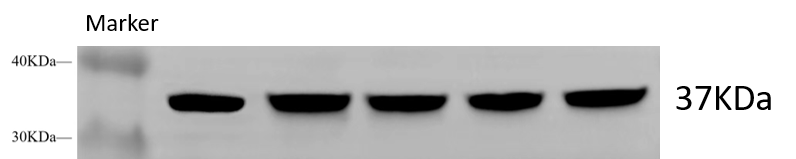


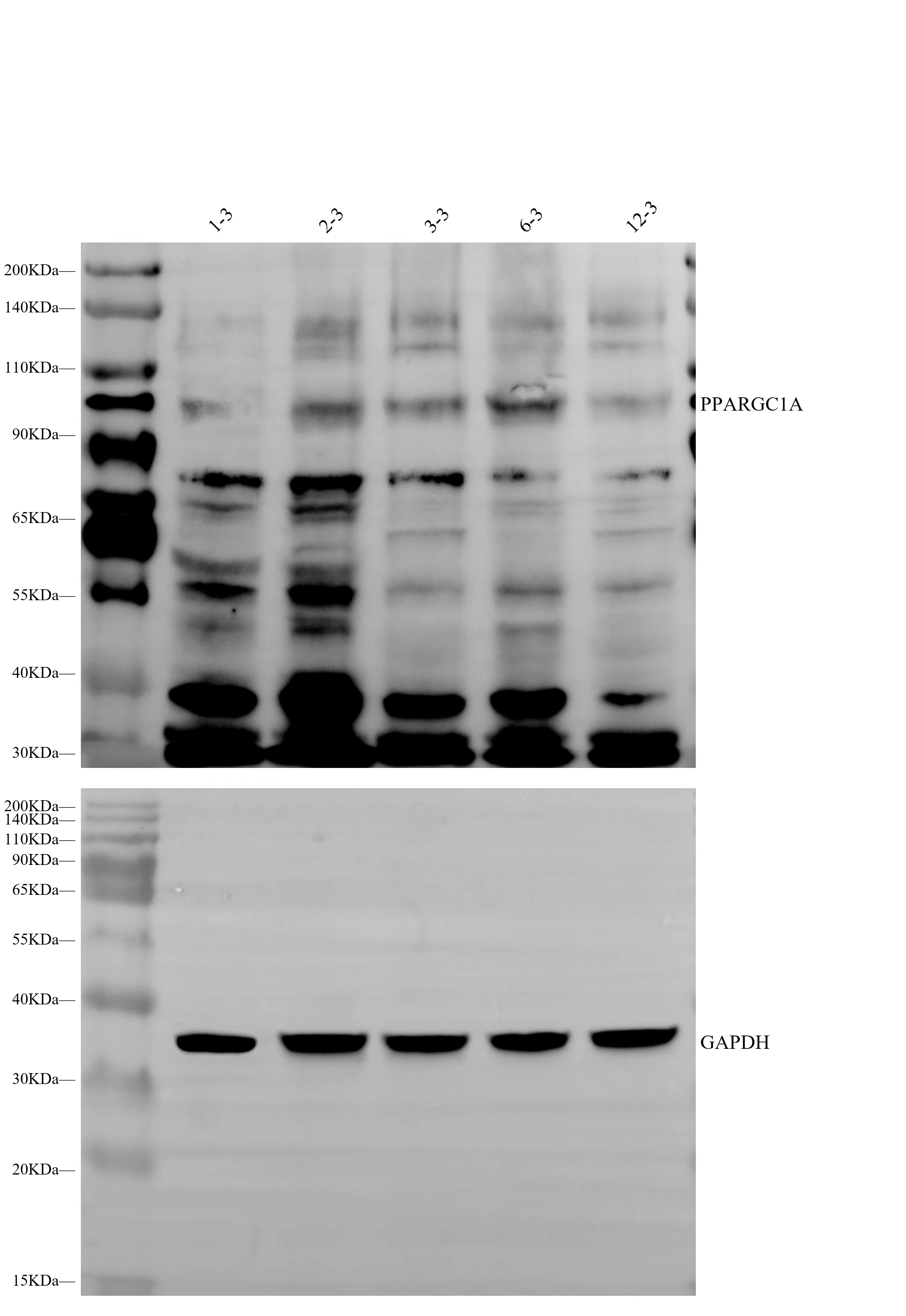


This is the corresponding internal reference image.

2. Adiponectin protein


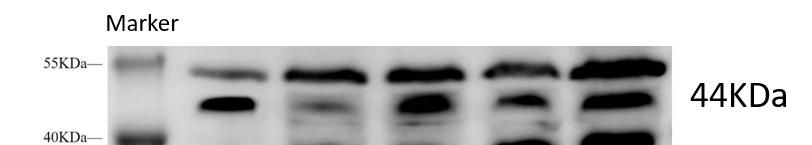


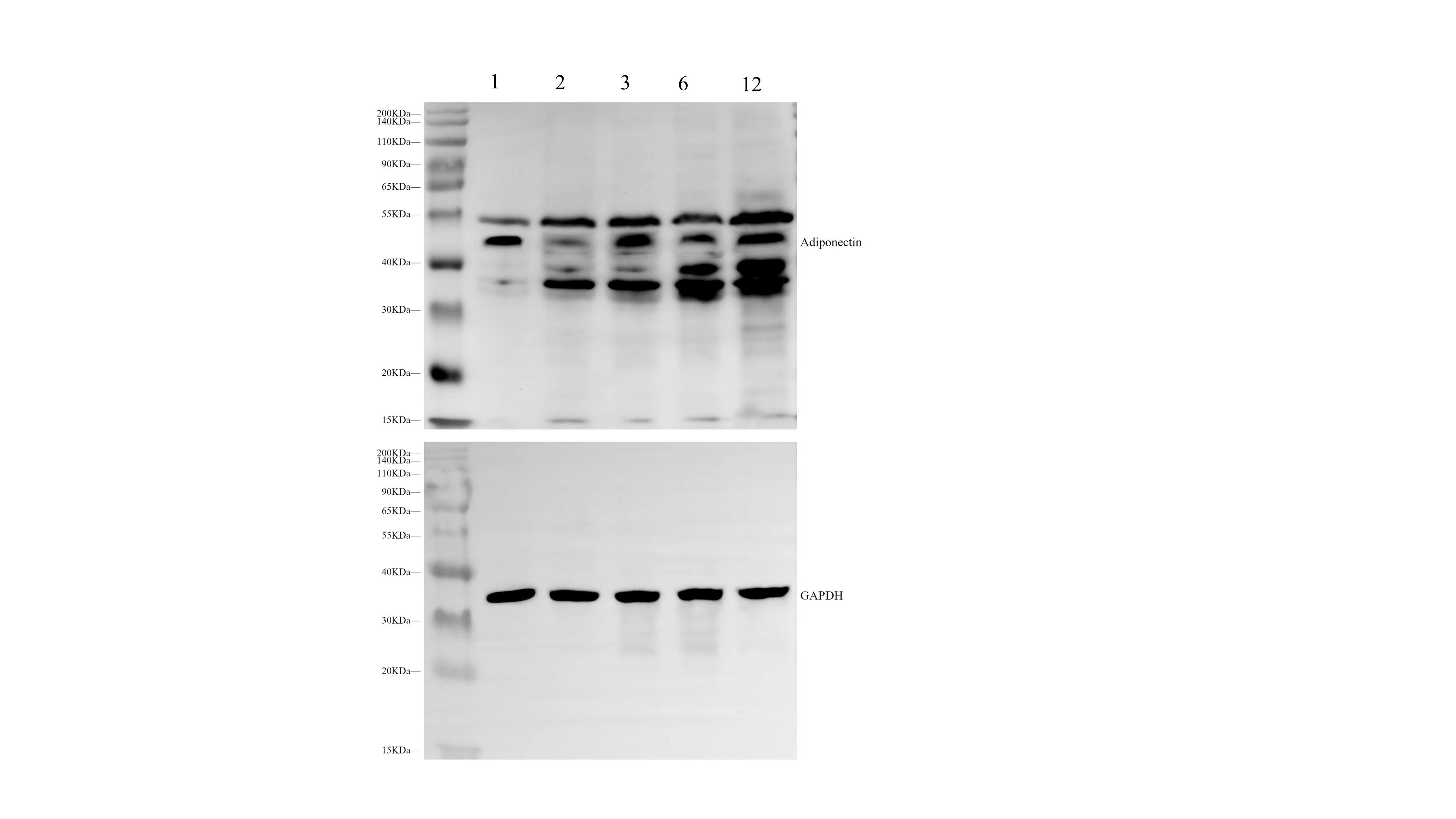


This image is a Western Blot image of Adiponectin protein.


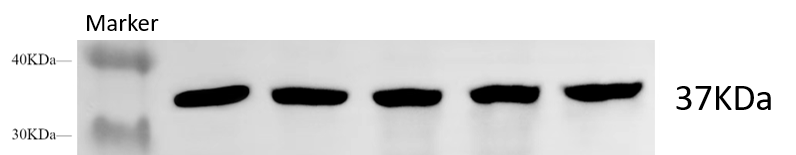


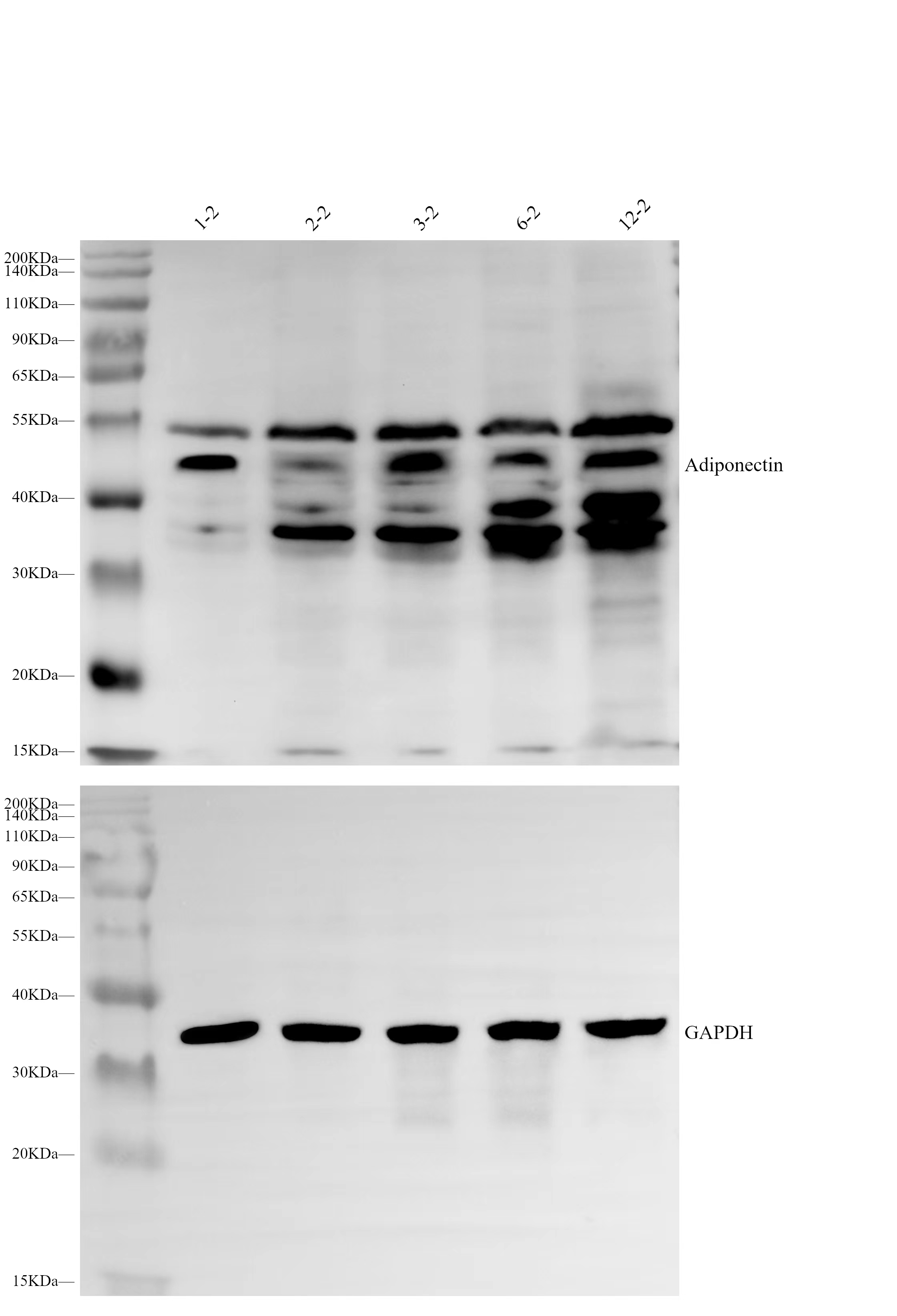


This is the corresponding internal reference image.
